# Features of Composite Layers Created Using an Aqueous Suspension of a Fluoropolymer

**DOI:** 10.3390/polym14214667

**Published:** 2022-11-01

**Authors:** Dmitriy Valerievich Mashtalyar, Konstantine Vakhtangovich Nadaraia, Evgeny Alekseevich Belov, Igor Mikhaylovich Imshinetskiy, Sergey Leonidovich Sinebrukhov, Sergey Vasilevich Gnedenkov

**Affiliations:** Institute of Chemistry, Far Eastern Branch of the Russian Academy of Sciences, 690022 Vladivostok, Russia

**Keywords:** composite coatings, plasma electrolytic oxidation, fluoropolymer, anti-corrosion protection, anti-wear protection, hydrophobic surface

## Abstract

This paper presents a method for the formation of composite-polymer-containing coatings on MA8 Mg alloy by plasma electrolytic oxidation (PEO), followed by the deposition of a fluoropolymer from an aqueous suspension of superdispersed polytetrafluoroethylene. The Scanning Electron Microscope(SEM), Energy Dispersive Spectroscopy(EDS), and X-ray Diffraction(XRD) analyses established morphological features as well as elemental and phase composition of composite coatings. The fact that the pores are filled with a fluoropolymer has been experimentally confirmed. An assessment of the corrosion properties of formed composite coatings revealed a decrease in the corrosion current density by more than four orders of magnitude in comparison with the base PEO layer. The highest resistance to the damaging effects of a corrosive environment, according to the results of long-term exposure tests, was demonstrated by coatings after three treatments with polytetrafluoroethylene. The obtained polymer-containing coatings have antifriction properties, reducing the wear of the coatings by more than 27-fold in comparison with the base PEO layer. It was revealed that composite coatings have superhydrophobic properties: the value of the contact angle reaches 154°, and the hysteresis of the contact angle is less than 10°.

## 1. Introduction

Prospects for the use of Mg alloys for various industries are determined by their high strength, low density, and high damping capacity [1,2,3]. These alloys have significant perspectives of application for the machine, aircraft, and rocket industries, as well as medicine, electronics, etc. [3,4,5,6,7]. However, due to low resistance to corrosion [1,8] and wear [9,10], the use of magnesium alloys is restricted.

The formation of protective coatings on the surface of magnesium alloys is a promising direction for protecting materials from aggressive environmental influences [11,12,13,14,15,16,17,18,19,20,21,22,23,24,25,26]. The use of plasma electrolytic oxidation (PEO) as a method for creating a durable, anticorrosive coating on the surface of metals and alloys was considered in [5,27,28,29,30,31,32,33], which presented the principles of formation and characteristics of these ceramic-like coatings.

The protective properties of PEO coatings are due to their chemical composition and morphological structure. Features of the formation of coatings by plasma electrolytic oxidation suggest the presence of outer porous and inner barrier layers. The porosity of PEO coatings varies from 5 to 50%, and the pore size changes from 0.01 to 10 µm [34,35,36,37,38,39,40,41,42,43,44,45,46]. Thus, the anticorrosive properties of coatings are determined by the characteristics of a dense barrier sublayer at the alloy/coating interface, namely its thickness, defectiveness, and composition. Moreover, in general, the thickness of the inner sublayer is much less than the outer porous one. At the same time, due to such a morphological structure, the PEO layer can serve as a good basis for the creation of composite coatings (CC), which make it possible to impart new functional properties to the treated surface, as was presented in [3,47,48,49,50,51,52,53,54,55,56,57,58,59,60,61,62,63,64,65,66,67,68,69,70]. In this case, the main promising approach is to fill the pores of the PEO coating with functional materials that have the necessary set of service characteristics. One such promising materials is polytetrafluoroethylene (PTFE), a chemically inert, electrically insulating material with antifriction properties and a very wide operating temperature range from −200 to +320 °C [71,72,73]. In this regard, the methods of forming composite coatings using PEO and fluoropolymer were considered in various papers [53,54,55,56,57,58,59,60,61,62,63,64,65,66,67,68,69,70,74,75,76,77,78,79,80].

One of the key factors affecting the properties of the composite coating is the method of applying PTFE to the preformed PEO layer. In previous works, the methods of triboelectric, electrophoretic deposition [81,82,83,84,85], as well as spraying or immersion [70,74,79,86,87,88] methods were studied. In all these papers, an isopropanol suspension of superdispersed polytetrafluoroethylene (SPTFE) of trademark “Forum^®^” was used. However, the use of an alcohol suspension is associated with certain technological limitations. Isopropyl alcohol, which was used as a dispersion medium for the formation of a suspension, is a highly volatile and flammable substance with a sharp unpleasant odor and an intoxicating narcotic effect. Its use requires special precautions to prevent possible fires or poisoning of personnel. In this regard, we have developed and applied an aqueous suspension of SPTFE. This article provides information on the described method of coatings formation, as well as on the properties of the resulting composite layers, depending on the cycles of processing PEO-coated samples in an aqueous suspension of SPTFE.

## 2. Materials and Methods

Magnesium alloy MA8 (Mg–Mn–Ce system, wt. %: 1.30 Mn; 0.15 Ce; Mg to the balance) was used as a substrate for the preparation of samples. The dimensions of the samples were 30 × 20 × 1 mm^3^. Before the coating formation, samples were subjected to mechanical processing with sanding paper with a gradual decrease in grain size to 30 μm. Then, the samples were washed with distilled water and alcohol in an ultrasonic bath for 5 min.

To prepare the suspension, as the dispersed phase in this work, we used superdispersed polytetrafluoroethylene. This organofluorine material was obtained by the method of thermogradient synthesis during the recycling of F–4 fluoroplast [89]. SPTFE powder contains particles ranging in size from 300 to 600 nm, with average size equal to 400 nm. Distilled water was used as a dispersion medium. The concentration of SPTFE in water was 20 wt.%. Polytetrafluoroethylene has hydrophobic properties, so it is impossible to obtain a stable suspension by simply mixing the components. Thus, to stabilize the obtained system and increase the wettability of SPTFE particles, the nonionic surfactant OP–10 (R_2_C_6_H_4_O(C_2_H_4_O)_10_) was added to the suspension at a concentration of 25 g l^− 1^.

On the basis of the previously developed principles of targeted synthesis of PEO layers on the surface of a substrate made of metals and alloys [33] and the conclusions obtained from the study of previously published works [16,90,91], in this work, in order to form PEO coatings, an electrolyte was used, containing 15 g l^−1^ sodium orthosilicate (Na_4_SiO_4_) and 5 g l^−1^ sodium fluoride (NaF). The surface of the samples was modified in two stages using a bipolar mode, in which anodic pulses alternated periodically with cathodic ones [33,80]. During the first stage, there is a potentiodynamic increase in the anode phase from 20 to 240 V, at a growth rate of 1.05 V s^−1^, with a stable cathode phase equal to −40 V. During the second stage, the anodic phase decreases at a rate of 0.07 V s^−1^ from 240 to 200 V; in the cathode phase, a potentiodynamic growth also occurred from −40 to −10 V, at a rate of 0.05 V s^−1^. The first stage lasts 200 s, and the second stage lasts 600 s. The ratio of the duration of the anodic and cathodic pulses was 1, the duty cycle was 50%, and the polarization frequency was 300 Hz. During the PEO, the electrolyte temperature (16 °C) was stabilized using a ChillerSmart H150–3000 cooling unit (LabTech Group, London, UK).

The formation of composite coatings was carried out by dipping (immersing) samples for 10 s in an aqueous suspension of a fluoropolymer, followed by drying in air under ambient conditions. For application of the composite layer, an RDC–21k device (Bungard, Leverkusen, Germany) was used, which allows the setting of the parameters for dipping samples into a suspension. Immersion occurred at a speed of 1 mm s^−1^ to a depth of 50 mm. The samples were withdrawn at a rate of 5 mm s^−1^. After complete withdrawal, the samples were dyed horizontally for a more even distribution of the polymer over the surface. Subsequently, the samples were heat treated at 315 °C for 15 min. Heat treatment was carried out after each deposition of the polymer to introduce it into the porous part of the coating [79,81,89]. In this work, the effect of the number of polymer layers on the properties of the formed composite coatings is presented for samples with one- (CC 1X), two- (CC 2X), and threefold (CC 3X) deposition of SPTFE.

The morphological features of the samples were studied by scanning electron microscopy (SEM) using the following equipment: Evex Mini–SEM (Evex Analytical Instruments, Belle Mead, NJ, USA) and EVO 40 (Carl Zeiss, Jena, Germany). The elemental composition of the coatings was studied using an INCA X–act energy dispersive spectroscopy (EDS) device (Oxford Instruments, Abingdon, UK).

The phase composition of the formed protective layers was determined at the Far East Center for Structural Research using a multipurpose Rigaku X-ray diffractometer (XRD, SmartLab, Tokyo, Japan) (CuKα radiation). The analysis was performed according to the Bragg–Brentano geometry in the angle range 2θ from 5° to 80° with a step of 0.02° and an exposure time of 1 s at each point. X-ray diffraction analysis was carried out using the “EVA” search program with the “PDF–2” database.

The electrochemical properties of magnesium alloy samples without treatment, with a PEO layer, and composite coatings were studied by the methods of potentiodynamic polarization and electrochemical impedance spectroscopy (EIS) using the VersaSTAT MC electrochemical system (Princeton Applied Research, Princeton, NJ, USA). The measurements were carried out using a three-electrode cell under standard conditions in a 3.5 wt.% NaCl solution. A niobium grid coated with platinum was used as a protoelectrode. A saturated calomel electrode (SCE) was used as the reference electrode; all potential values given in this work are relative to this electrode. The working surface area of the samples was 1 cm^2^. Before the start of electrochemical measurements, the samples were kept in solution for 30 min to reach a steady state condition at the studied sample/electrolyte interface. During exposure, the electrode potential was fixed; the last measured value was stabilized potentiostatically during EIS. During the recording of the impedance spectrum, the sinusoidal signal had an amplitude of 10 mV (rms). The studies were carried out in the frequency range from 0.01 Hz to 1 MHz with a logarithmic sweep of 10 points per decade. Additionally, the OCP of the coatings was measured in a separate experiment lasting 50 min.

For a detailed study of changes in the properties of the obtained coatings in a 3.5 wt.% NaCl, the samples were kept in an electrolyte at the standard conditions for 72 h with the recording of impedance spectra after 1, 24, and 72 h in the same frequency range but with a logarithmic sweep of 7 points per decade.

The potentiodynamic polarization measurements were carried out at a potential sweep rate of 1 mV s^−1^ in the range from *E*_C_ – 0.15 V to *E*_C_ + 0.50 V. To describe the experimental dependence of the current density *I* on the potential *E*, the Levenberg–Marquardt method was used (Equation (1)):

(1) $$I=I_{C}\left(10^{\frac{E-E_{C}}{\beta_{a}}}+10^{-\frac{\left(E-E_{C}\right)}{\beta_{c}}}\right).$$

This method makes it possible to obtain the most accurate calculated values of parameters such as corrosion potential *E*_C_, corrosion current density *I*_C_, as well as the slopes of the cathodic *β*_c_ and anodic *β*_a_ polarization curves.

The polarization resistance *R*_P_ was determined in a separate experiment with the potentiodynamic polarization of the sample in the potential range Δ*E* = *E*_C_ *±* 20 mV with a sweep rate of 0.167 mV s^−1^, in which a linear dependence *I = f* (*E*) is observed. *R*_P_ was calculated using the following Equation (2):

(2) $$R_{P}=\frac{\Delta E}{\Delta I}.$$

The wear resistance of coatings was determined on a TRB–S–DE device (CSM Instruments, Peseux, Switzerland). The tests were carried out according to the “ball–disk” scheme at the standard conditions with the sliding speed of 50 mm s^−1^ until the coating was completely abraded in the dry friction mode under the action of a force of 10 N. The track of the counterbody movement along the sample was a circle with a diameter of 10 mm. A corundum ball (α–Al_2_O_3_) with a diameter of 10 mm was used as a counterbody. Surface wear was measured using a Surtronic 25 profilometer (Taylor Hobson Ltd., Leicester, UK).

From these data, the surface wear rate was calculated by Equation (3):

(3) $$P=\frac{\Delta V}{NF},$$

where *P* is the wear rate value (mm^3^ (N m)^−1^), Δ*V* is the worn volume (mm^3^), *N* is the distance moved (m), and *F* is the normal load (N).

(4) ΔV=SL,

where *L* is the length of the of the wear track (mm), *S* is cross-section area of the wear track (mm^2^).

Wear of the counterbody, during all tests, was not detected and was not considered in the calculation.

The adhesion properties of coatings were evaluated using a Revetest Scratch Tester (CSM Instruments, Peseux, Switzerland). The study of adhesion was carried out by measuring the critical load at which specific coating failures were observed. The indenter was a conical diamond tip (Rockwell) with a tip angle of 136° and a curvature radius of 200 µm. The indenter formed a line 5 mm long on the surface of the sample, with an increase of the applied load from 1 to 20 N at a rate of 0.1 N s^−1^.

The wettability of obtained coatings was studied by the sessile drop method using a DSA100 device (Krüss, Hamburg, Germany) according to the technique described in [92,93]. The test liquid was distilled water. The drop volume was 10 μl. During calculation of the contact angle, the Young–Laplace method was used to take into account the distortion of the drop shape under the gravity.

Advancing and receding contact angles were measured according to the method described in [94]. The contact angle hysteresis was calculated as the difference between the advancing contact angle and the receding contact angle.

## 3. Results and Discussion

### 3.1. Morphology and Composition of Coatings

**Figure 1:**
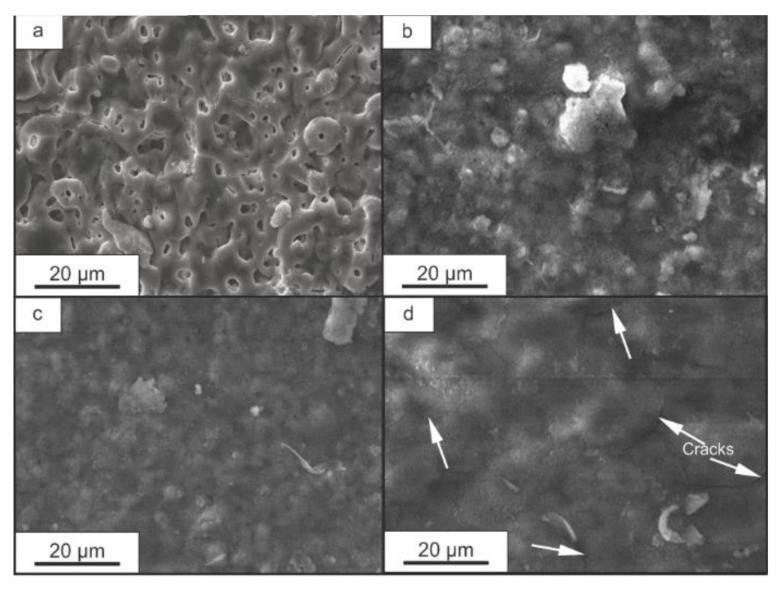
SEM images of the surface of samples with PEO coating (**a**), CC 1X (**b**), CC 2X (**c**), and CC 3X (**d**). Arrows indicate cracks.

The properties of the composite coatings studied in this work, which were formed at different multiplicities of treatment of a specimen with a base PEO layer in an aqueous suspension of SPTFE, are due to a change in the morphological structure of the coating as well as the filling of its pores with a fluoropolymer. An analysis of the surface of composite coatings shows that with a single application of the polymer, the number of visible pores and defects decreased sharply (from 37% to 3%) compared with the base PEO layer (Figure 1a,b). A further application of SPTFE led to the formation of a more uniform surface of coatings with high homogeneity (Figure 1c,d). During the formation of composite layers, stresses appeared in the polymer material, which did not have time to relax and led to the formation of microcracks in the surface (Figure 1b–d). At the same time, the presence of cracks in the polymer film did not greatly affect the protection properties of the formed coatings.

**Figure 2:**
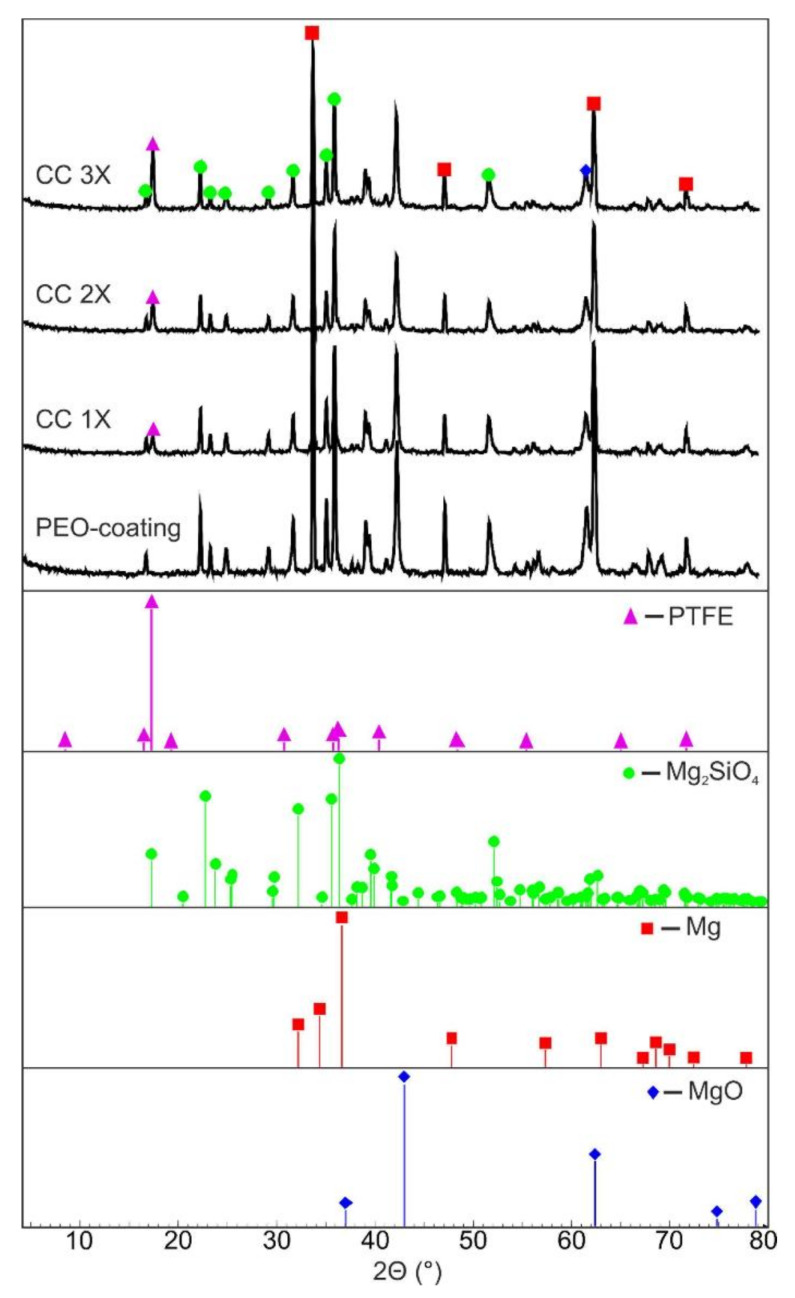
XRD patterns of samples with a base PEO coating, CC 1X, CC 2X, and CC 3X.

According to XRD data, the PEO coating contains periclase (MgO JCPDS Card No: 00–045–0946) and forsterite (Mg_2_SiO_4_ JCPDS Card No: 01–084–1402) (Figure 2). The presented compounds were formed from electrolyte components and a metal substrate in the process of PEO under the action of microdischarges [33]. The diffraction pattern of the composite coating with a single application of a fluoropolymer (Figure 2) contains PTFE (JCPDS Card No: 01–047–2217) peaks, which indicate the incorporation of the polymer into the PEO coating during the formation of composite layers.

An increase in the XRD spectrum (Figure 2) of the intensity of one of the peaks associated with polytetrafluoroethylene, in the region 2Θ = 17–18°, was associated with an increase in the amount of the crystalline phase in the surface layer.

**Figure 3:**
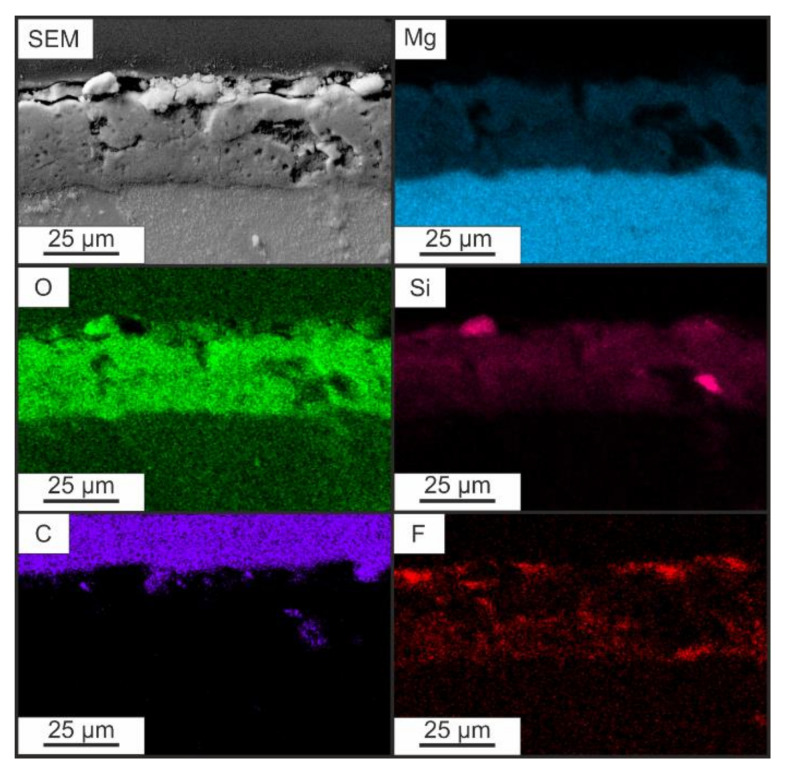
SEM image of a cross-section of a composite coating with EDS mapping of elements along the coating thickness.

The distribution of elements over the thickness of the composite coating (CC 3X) was studied by the EDS of the cross-section (Figure 3). The relatively uniform distribution of elements such as Mg, Si, and O over the coating’s thickness is the result of the interaction of the metal substrate and the electrolyte during plasma electrolytic treatment. The distribution of SPTFE components C and F over the thickness of the CC 3X indicates the filling of the pores of the PEO layer and the formation of an additional protective barrier layer on its surface (Figure 3). Thus, the formed composite coating isolates the metal substrate from the environment.

### 3.2. Electrochemical Studies

**Figure 4:**
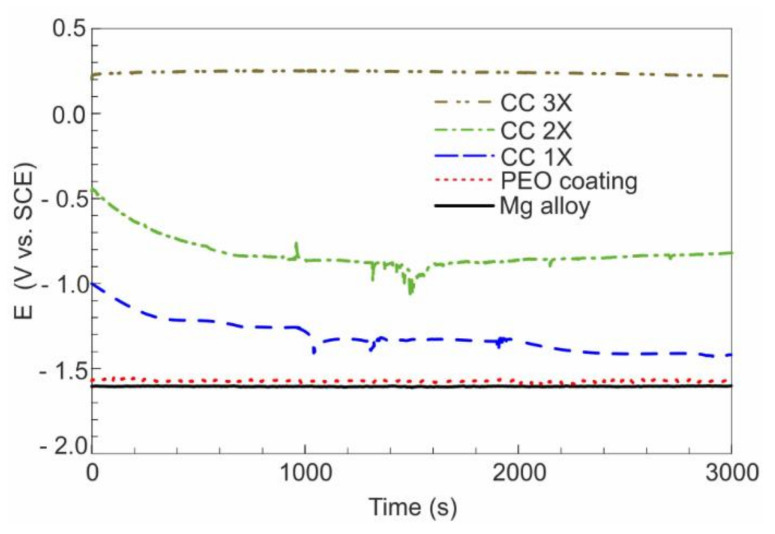
Dynamics of changes of the corrosion potential during 50 min exposure in 3.5 wt.% NaCl solution for samples with various types of surface processing.

Figure 4 shows the change in the electrode potential E of the samples under the study during immersion in a 3.5 wt.% NaCl solution for 50 min.

An uncoated magnesium alloy demonstrates relatively stable electrode potential values of approximately −1.65 V during the experiment time (Figure 4). In this case, an oxide/hydroxide layer is constantly formed and dissolved on the surface of alloy in an aggressive environment.

The electrode potential of the PEO-coated sample is approximately −1.6 V and practically does not change during exposure to 3.5 wt.% NaCl (Figure 4). For samples with composite coatings, the electrode potential increases with an increase in the amount of fluoropolymer deposited on the surface (Figure 4) and is significantly higher compared with the potential for an uncoated sample and a sample with a PEO coating. At the same time, the nature of the change in the electrode potential of samples with CC 1X and CC 2X differs from the behavior of CC 3X. The potential of the sample with the CC 1X gradually decreases to a value of −1.5 V within 50 min. This behavior of the electrode potential is due to the insufficient continuity of the polymer film (Figure 1b) and incomplete filling of pores and defects, through which the aggressive medium has access to the substrate. A similar behavior of the electrode potential over time is observed for a sample with a CC 2X, for which a drop in potential from −0.5 V to −0.8 V was recorded during the first 10 min, with further stabilization to values of approximately −0.8 V. However, the more uniform surface structure of CC 2X (Figure 4) led to the higher electrode potential and faster stabilization relative to CC 1X (Figure 4).

The highest electrode potential was recorded for the sample with the CC 3X. Stable potential values of approximately 0.25 V are observed throughout the entire time of the experiment, in contrast with other samples with composite coatings (Figure 4). This behavior of the electrode potential is an aftermath of the full filling of the porous part (pores and defects) of the PEO layer with polymer and the formation of a continuous polymer-containing layer (Figure 1d and Figure 4).

**Figure 5:**
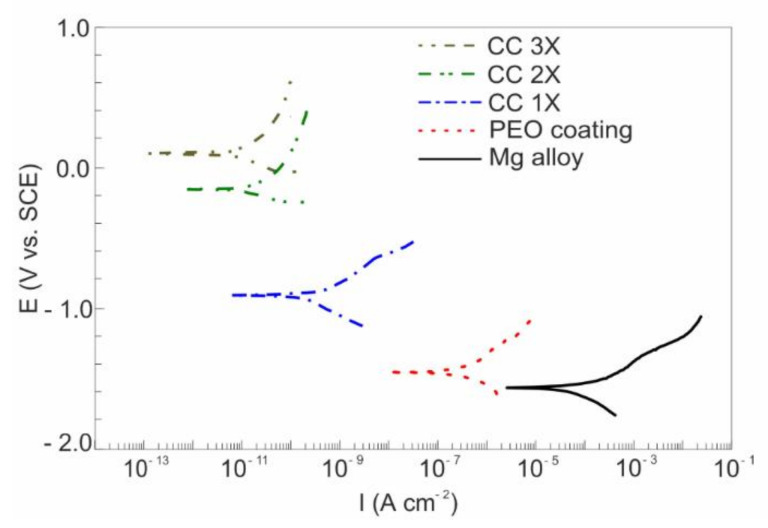
Polarization curves of samples with different processing methods.

**Figure 6:**
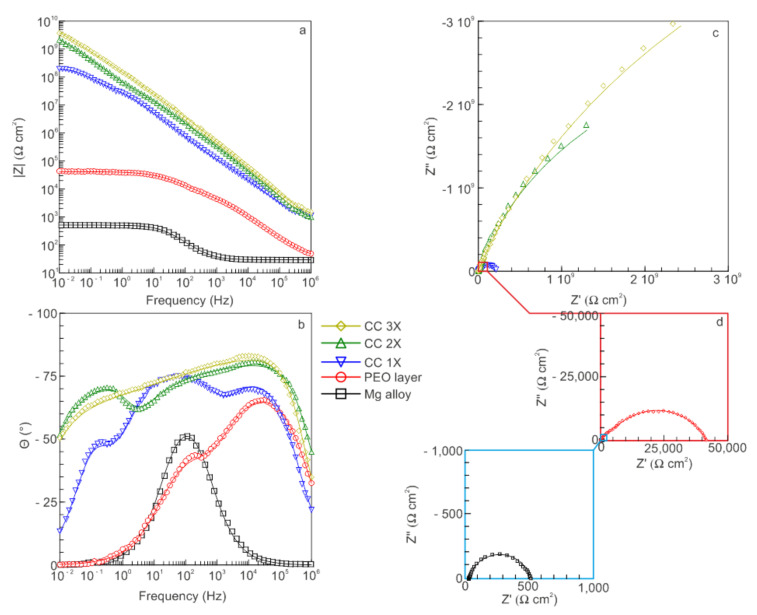
Bode plots (dependence of the impedance modulus |Z| (**a**) and phase angle θ (**b**) on frequency f) and Nyquist plots (dependence of the imaginary part of the impedance Z″ on the real part of the impedance Z′) (**c**,**d**) obtained for bare alloy, PEO layer, and composite coatings. The symbols represent the experimental data, and the lines are the fitting curves calculated according to the EEC.

The resistance of coatings to corrosion was studied by the methods of potentiodynamic polarization (Figure 5) and EIS (Figure 6), and the main results are presented in Table 1. An analysis of the presented data shows that the PEO coating itself increases the corrosion resistance by two orders of magnitude compared with uncoated Mg alloy (Table 1). Composite coatings, on the other hand, made it possible to reduce the corrosion current density I_C_ by more than three orders of magnitude relative to the PEO layer without modification.

**Figure 7:**
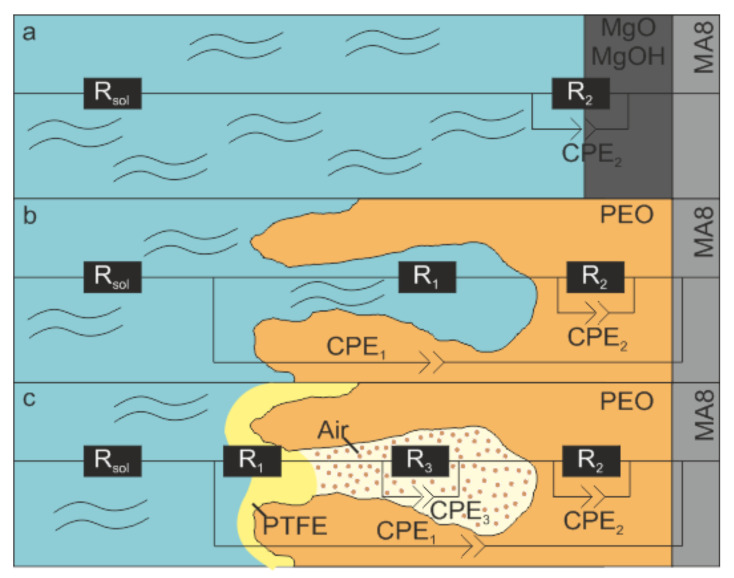
Modeling of surface layers and corresponding equivalent electrical circuits obtained by fitting experimental data on impedance: (**a**) magnesium alloy with single R–CPE chain, (**b**) PEO coating with two R–CPE chains, and (**c**) three R–CPE chains for composite coatings.

The spectra obtained by EIS are presented in Figure 6 in the form of Bode plots (representing the dependence of the impedance modulus |Z| and phase angle on the frequency f) and Nyquist plots (representing the dependence of the imaginary part of the impedance Z″ on its real part Z′). The experimentally obtained data are presented in Figure 6 as symbols, and the fitted curves describing the spectra based on the corresponding equivalent electrical circuits (Figure 7) are presented by solid lines.

This is confirmed by the analysis of the calculated parameters of the elements of EEC (Table 2), obtained using Equation (5), where Q is a pre-exponential factor, a frequency independent parameter; i = √ (−1) is an imaginary unit; ω = 2πf is an angular frequency; and n is an exponential coefficient that determines the nature of the frequency dependence (−1 ≤ n ≤ 1).

To correctly interpret the EIS data, a constant phase element (CPE) was used instead of an ideal capacitance element. The heterogeneity of the coating system leads to the fact that in the analysis of electric equivalent circuits (EEC) it is necessary to apply CPE. The CPE impedance can be calculated using Equation (5):

(5) $$Z_{\mathrm{CPE}}\left(\omega \right)=\frac{1}{Q{\left(i\omega \right)}^{n}}.$$

On the basis of the analysis of the obtained EIS data, it can be seen that the formation of a ceramic-like PEO layer on the surface of the Mg alloy led to an increase in the values of the impedance modulus at low frequencies |Z|_f=0.01 Hz_ by approximately two orders of magnitude compared with the bare alloy. The formation of a composite-polymer-containing coating made it possible to increase |Z|_f = 0.01 Hz_ by more than four orders of magnitude compared with the PEO coating. Each subsequent processing with SPTFE increased the impedance modulus 1.5–2-fold (Table 1).

The obtained values of the impedance modulus for the sample with CC 3X are shown to be the largest (Table 1). For the sample after three SPTFE treatments, the value |Z|_f = 0.01 Hz_ = 1.14 10^9^ Ω cm^2^, which exceeds the results obtained for uncoated samples and PEO-coated samples by more than six and five orders of magnitude, respectively. Such a significant increase in the impedance modulus for the composite coating in comparison with the base PEO coating is a consequence of sealing pores and defects of the base PEO layer with the organofluorine material (Figure 1, Figure 6 and Figure 7).

According to the results of the electrochemical modeling performed, the spectrum for the uncoated sample (Figure 6) can be fitted using a simplified EEC with one R_2_–CPE_2_ chain (Figure 7), where R_2_ is a charge transfer resistance, and CPE_2_ is a double layer capacitance (Table 2). These conclusions are based on the analysis of the Bode plot for bare Mg alloy: there is only one maximum (time constant) at the middle frequency region of phase angle dependence on the frequency f (Figure 6).

For a sample with a base PEO coating, the dependence of the phase angle on frequency has two bends (time constants). Thus, the experimental spectrum can be described by a two-series-parallel-equivalent electrical circuit shown in Figure 7, in which the R_2_–CPE_2_ chain models the charge transfer through the non-porous sublayer of the PEO coating, and the R_1_–CPE_1_ chain models the charge transfer through its porous part. Note that the CPE_1_ element also describes the geometric capacitance of the PEO layer (Figure 7).

The dependence of the phase angle on frequency for composite coatings shows several bends that determine the choice of the appropriate EEC modeling of the experimental data (Figure 6 and Figure 7). The spectra of all samples with composite coatings can be fitted by an EEC containing three chains, in which the elements CPE_1_ and R_1_ are responsible, respectively, for the geometric capacitance of the entire coating and the resistance of a thin film of polymer material on top and in the pores of the composite layer (Figure 7). The R_2_–CPE_2_ chain describes the behavior of a dense non-porous sublayer of the composite coating, and R_3_–CPE_3_ is responsible for the pseudo-layer of air trapped in the pores of the PEO coating and sealed on top with a polymer plug (Figure 7).

An analysis of the impedance spectra of samples with CC allows us to conclude that the electrode/electrolyte interface has capacitive character for such type of coatings, which indicates high protective properties of the formed layers. Obviously, the properties of the composite coatings noted above are from such a feature as the penetration and sealing of pores and defects by SPTFE.

A decrease in Q_1_ (parameters characterizing the porous part of the coatings) for composite layers in comparison with PEO coatings are the result of constriction (reduction of cross-section) of the remaining pores and increasing the thickness of the CC after SPTFE treatment. Note that there is an increase in the resistance R_1_ for CC 2X and CC 3X in comparison with CC 1X (Table 2). Taking into account the values of the coefficient n_1_ (0.54) for CC 1X and the results of SEM data on the continuity of the coating (Table 2, Figure 1b), it can be concluded that some diffusion processes occur during electrochemical tests. The R_1_ values for CC 2X and CC 3X are quite close and do not exceed 1.7 10^3^ Ω cm^2^ (Table 2). Thus, the contribution of the outer thin polymer film to the protective properties of the composite coating is small. This assumption is also confirmed by the values of n_1_ for CC 2X and CC 3X: 0.70 and 0.60, respectively (Table 2). The observed trend toward an increase in the resistance of the non-porous sublayer R_2_ and a decrease in the pre-exponential factor Q_2_ in the second time constant is the result of an increase in the thickness of the non-porous sublayer due to the deposition of PTFE on the bottom of the pores during the formation of the composite coating. High values of the electrical resistance R_3_ and low values of the pre-exponential factor Q_3_ for composite coatings characterize the conductivity and the thickness of the air trap located between the non-porous sublayer and the polymer plug.

**Figure 8:**
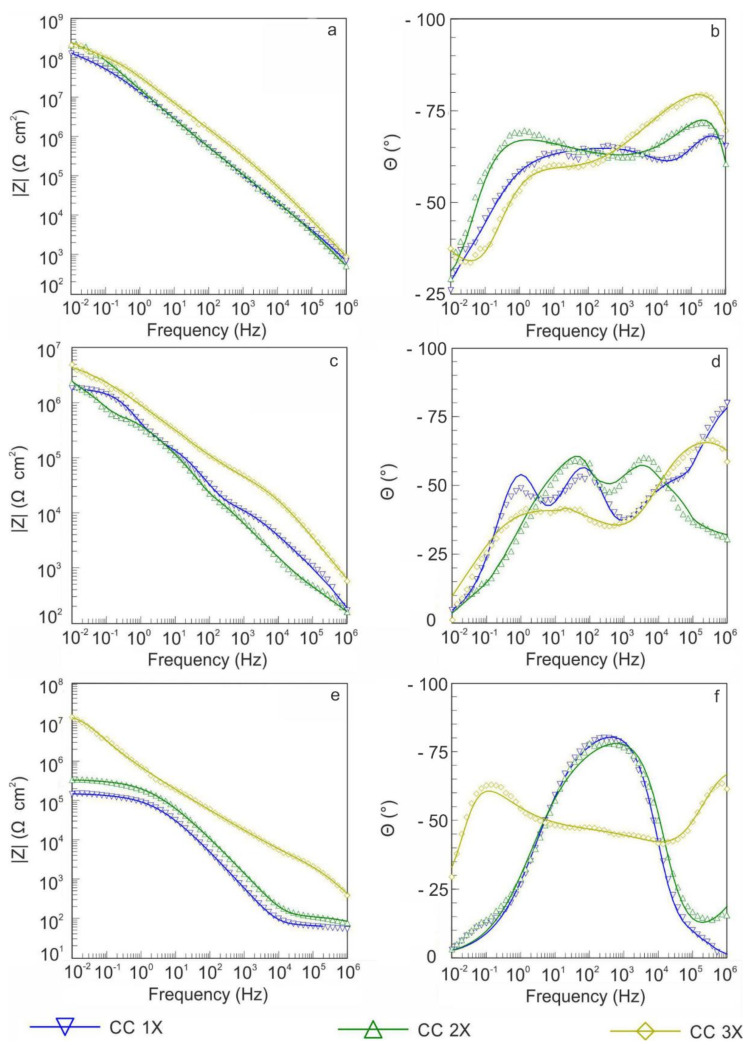
Graphs of change of the impedance modulus |Z| and phase angle depending on frequency, presented as Bode plots, obtained by immersing samples with composite coatings in 3.5 wt.% NaCl solution (1 h (**a**,**b**), 24 h (**c**,**d**), 72 h (**e**,**f**)). Symbols correspond to measurement data, and lines correspond to curves obtained by approximation and calculation.

To study the corrosion resistance of the obtained composite layers during long-term contact with an aggressive medium, the samples were kept in a 3.5 wt.% NaCl solution for 72 h, with the recording of the impedance spectrum after 1, 24, and 72 h of exposure. On the basis the presented data (Figure 8, Table 3), it can be concluded that impedance modulus for composite coatings decreased by one order of magnitude during 1 h of exposure compared with exposure of 0.5 h (spectra for composite layers after 0.5 h exposure in electrolyte are presented in Figure 6). The |Z|_f = 0.01 Hz_ after 24 h of testing was reduced compared with the values obtained during the first 1 h of experiment (Figure 6 and Figure 8). After 72 h, the protective properties of composite coatings decreased, and the observed behavior of the dependence of phase angle on frequency for samples with CC 1X and CC 2X was similar to the behavior of metal with a layer of corrosion products (Figure 6 and Figure 8). However, the |Z|_f = 0.01 Hz_ for these coatings were higher than that of the base PEO coating after 0.5 h of immersion in corrosive media (Figure 6 and Figure 8, Table 1, Table 3). This, in turn, indicates the high anti-corrosive properties of the CC 1X and CC 2X. Given these values of the impedance modulus and the behavior of the phase angle (Figure 8), it can be concluded that certain changes occurred in the structure of the coatings. Apparently, the electrolyte, penetrating through incompletely sealed pores, interacts with the substrate metal, resulting in the formation of corrosion products. Thus, the nature of the charge transfer is no longer determined by the composite layer but by the interaction of the magnesium alloy with the electrolyte (Figure 8). However, the area of such interaction is small, which caused the obtained data of the impedance modulus for CC 1X and CC 2X to exceed the values for uncoated metal by two orders of magnitude (Figure 6 and Figure 8, Table 1, Table 3).

Also of interest is the increase in the performance of the |Z|_f = 0.01 Hz_ after 72 h of the experiment for CC 3X (Figure 8, Table 3). On the basis of the analysis of SEM images, it can be concluded that the electrolyte can penetrate through a small number of cracks (Figure 1d) to the substrate material. Formed corrosion products filled the pores and blocked access to the substrate. Furthermore, since the number of defects on the surface of CC 3X is extremely small, after formation of corrosion products at the bottom of the through pores, the values of the impedance modulus increased (Figure 8, Table 3).

Long-term immersion in a 3.5 wt.% NaCl solution had a serious impact on the formed composite coatings. The presence of even the smallest pores leads to the penetration of an aggressive medium to the substrate metal, the formation of corrosion products, the undermining of the protective coating, and the formation of fracture centers. This leads to severe degradation of the protective coating. The destruction of the coatings leads to a decrease of the impedance modulus. However, after 72 h of exposure, CC demonstrated the values of protective properties much higher than those of untreated Mg alloy or base PEO coating (Figure 6 and Figure 8); thus, the protective resource of this type of composite coatings is enough for long-term protection of substrate from the severe corrosion conditions

### 3.3. Wear Resistance of Coatings

**Figure 9:**
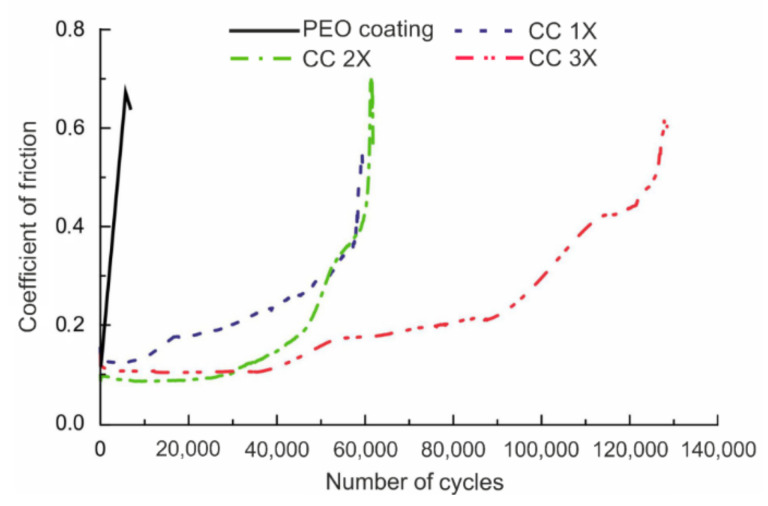
Dependence of the coefficient of friction on the number on the number of cycles for samples with different types of surface treatment.

From an analysis of the results of tribological tests, presented as a dependence of the coefficient of friction on the number of wear cycles (Figure 9), it can be concluded that PTFE makes a significant contribution to the wear resistance of composite layers, as well as to the wear process itself. On the basis of the data, we note that the base PEO layer was destroyed after ~2 200 cycles (Figure 9). From the graph analysis, it can be concluded that there is a sharp increase in the friction coefficient from the beginning of the tests, which indicates a rapid and uniform destruction of the porous part of the PEO layer and its further abrasion. (Figure 9). When applying a polymer material to the surface of the coating, the wear changes.

Thus, for composite coatings CC 1X, two stages of wear are observed, and the features of these stages differ. The first stage is associated with abrasion of the outer part of PEO coating matrix with PTFE embedded in it (Figure 9). The duration of this stage is approximately 60 000 cycles. Then, there is a sharp, almost linear rise in the values of the coefficient of friction. At this stage, the coating is completely abraded down to the metal.

The wear features of CC 2X is distinguished by the presence of three stages (Figure 9). The first stage is a consequence of the presence of a polymer film on the surface of the composite coating, which reduces the coefficient of friction (Figure 9). Further, starting from ~50,000 cycles, the stage of wear of the composite-polymer-containing (porous outer part) layer begins. Then, as in the case of CC 1X, a sharp rise in the coefficient of friction is observed, associated with the complete destruction of the inner part of the coating not containing polymer (Figure 9).

For CC 3X, it is possible to distinguish four stages of wear (Figure 9). The first of them, as well as for CC 2X, is due to friction between the counterbody and the thin polymer film on the surface of the CC (Figure 9). The second stage is a consequence of the partial destruction of the polymer film during friction and the interaction of the counterbody with the hard PEO- layer, which is also indicated by an increase in the coefficient of friction (Figure 9). Next, there is a stage of gradual abrasion of the composite coating. Note that the duration of this stage is much longer than that for CC 2X (Figure 9). The last stage, as in the case of other polymer-containing layers, is due to the complete destruction of the coating to the metal.

From the data obtained (Table 4), it was found that the introduction of a fluoropolymer into the porous layer of the base PEO coating reduces the level of surface wear by 1.7 times. Samples with the highest level of wear resistance are CC 3X; for these polymer-containing layers, wear was reduced more than 28-fold in comparison with the base PEO coating (Table 4).

Thus, it can be noted that the introduction of SPTFE into the porous structure of the coating affects not only the wear resistance but also the nature of abrasion, depending on the amount of PTFE in the composition of the coatings (Figure 9).

### 3.4. Coatings Adhesion

**Figure 10:**
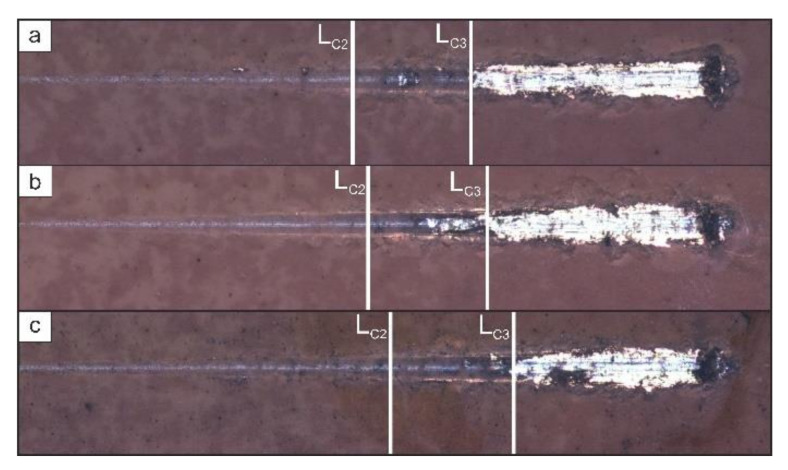
Optical images of scratches on the surface of CC 1X (**a**), CC 2X (**b**), and CC 3X (**c**).

An analysis of the adhesive properties of samples with different types of coatings enables one to conclude that the fluoropolymer material has a positive effect on coatings adhesion (Figure 10).

The load L_C2_, at which partial delamination of the coating is observed, for samples with a polymer-containing layer increased by more than 40% relative to the base PEO coating (Table 4). The phenomenon for such an increase in the applied load can be explained by the influence of the fluoropolymer material, which, during the penetration process, filled the pores with defects, thereby making the coating more continuous and distributing the load more evenly over the entire surface.

The value of L_C3_ (Figure 10, Table 4) at which the coating is destroyed down to the substrate, is more than 20% higher for composite layers than for the base PEO layer. This is due to the presence of SPTFE in the outer porous part of the composite coating: the polymer acts as a dry lubricant due to its low coefficient of friction. L_C3_ fluctuates within 10% for composite layers due to the difference in the amount of applied fluoropolymer. This is caused by the filling of pores and defects in the porous sublayer through which the indenter passes during its course, which leads to the complete destruction of the protective layer under different loads.

### 3.5. Coating Wettability Test

**Figure 11:**
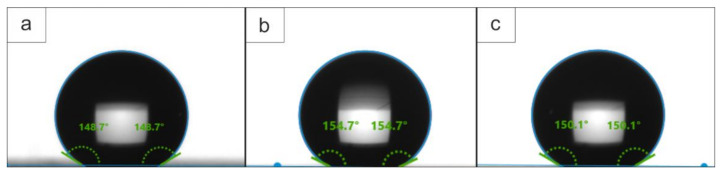
Optical images of a sessile drop on the surface of coatings with (**a**) CC 1X, (**b**) CC 2X, and (**c**) CC 3X. The difference in the CA values presented in the figure and those in Table 5 is due to the use of average values of several CA measurements in Table 5.

According to wettability tests, the coating with PTFE applied to its surface showed higher hydrophobic properties than the unmodified PEO layer. Thus, after a single treatment with a fluoropolymer of a hydrophilic PEO coating, the resulting composite coating acquired hydrophobic properties. The contact angle (CA) reached values of 149° (which is 100° more than that of the base PEO coating) (Table 5, Figure 11). For CC 2X, the CA reached 157°. With a further increase in the multiplicity of application of the fluoropolymer material, there was a reduction in CA (Table 5), which was caused by a change in the surface structure, a decrease in its roughness, and the appearance of a more uniform layer morphology (Figure 1c,d).

Superhydrophobic materials are characterized by a surface contact angle of more than 150° and a low contact angle hysteresis not exceeding 10° [94]. For CC 2X and CC 3X, the contact angle exceeded 150°. However, only for CC 2X, the contact angle hysteresis is less than 10° (Table 5), which makes it possible to characterize the resulting coating as superhydrophobic. The superhydrophobic properties are due to the presence of an irregular hierarchical structure on the surface of CC 2X.

## 4. Conclusions

A method for formation of composite-polymer-containing coatings on the MA8 magnesium alloy was created, which consists of processing the sample by plasma electrolytic oxidation followed by application of superdispersed polytetrafluoroethylene from an aqueous suspension.

The obtained coatings reduce the corrosion current density by six orders of magnitude in comparison with uncoated metal and three orders of magnitude in comparison with the base PEO layer.

Composite-polymer-containing coatings significantly improve the tribological properties of a surface layer of the MA8 magnesium alloy. Wear of material compared with PEO coating is reduced 27-fold.

The embedding of SPTFE from an aqueous suspension into composite coatings makes it possible to impart superhydrophobic properties to the surface, providing a high contact angle (more than 150°) and a contact angle hysteresis lower than 10°.

## Figures and Tables

**Table 1:** Corrosion characteristics (corrosion potential E_C_, corrosion current density I_C_, polarization resistance R_P_, and impedance modulus measured at low frequencies |Z|_f = 0.01 Hz_,) of Mg alloy samples with various types of coatings in 3.5 wt.% NaCl solution.

Sample	*E*_C_ (V)	*R*_P_ (Ω cm^2^)	*I*_C_ (A cm^−2^)	*|Z|_f_*_= 0.01 Hz_ (Ω cm^2^)
Without coating	−1.56	489.5	3.3 10^−5^	717.7
With PEO coating	−1.43	1.1 10^5^	2.4 10^−7^	6.3 10^4^
CC 1X	−0.91	2.2 10^8^	2.5 10^−10^	1.3 10^8^
CC 2X	−0.18	2.9 10^8^	1.6 10^−10^	1.8 10^9^
CC 3X	0.12	6.3 10^8^	7.7 10^−11^	2.8 10^9^

**Table 2:** Calculated parameters of the equivalent electrical circuits for the sample with various types of surface treatment in 3.5 wt. % NaCl solution.

Sample	R_1_ (Ω cm^2^)	CPE		R_2_ (Ω cm^2^)	CPE		R_3_ (Ω cm^2^)	CPE	
		*Q*_1_ (Ω ^−1^ cm^−2^ c^n^)	*n_1_*		*Q*_2_ (Ω ^−1^ cm^−2^ c^n^)	*n*_2_		*Q*_3_ (Ω ^−1^ cm^−2^ c^n^)	*n*_3_
Mg alloy	–	–	–	477	3.32 10^−5^	0.85	–	–	–
PEO coating	8.9 10^3^	2.1 10^−7^	0.76	3.3 10^4^	9.9 10^−7^	0.65	–	–	–
CC 1X	1.1 10^3^	7.4 10^−9^	0.54	9.9 10^3^	3.6 10^−9^	0.98	2.4 10^8^	4.8 10^−9^	0.85
CC 2X	1.7 10^3^	2.5 10^−9^	0.70	3.2 10^6^	4.2 10^−10^	0.99	10^10^	1.2 10^−9^	0.99
CC 3X	1.6 10^3^	10^−9^	0.60	4.2 10^6^	9.7 10^−10^	0.94	1.2 10^10^	7.2 10^−10^	0.98

**Table 3:** Changes in the impedance modulus at a frequency of 0.01 Hz, when samples were kept for 1, 24, and 72 h in a 3.5 wt.% NaCl solution.

Sample	*|Z|_f_*_= 0,01 Гц_ (Ω cm^2^)		
	1 h	24 h	72 h
CC 1X	1.43 107	3.33 105	1.46 105
CC 2X	2.08 108	1.41 106	3.36 105
CC 3X	2.1 108	9.57 106	1.31 107

**Table 4:** Wear and adhesive properties of specimens from magnesium alloy MA8.

Sample	Wear (mm^3^ N^−1^ m^−1^)	L_C2_ (N)	L_C3_ (N)
With PEO coating	1.7 10^−3^	4.6	10.2
CC 1X	9.9 10^−4^	6.7	11.9
CC 2X	2.0 10^−4^	6.8	12.9
CC 3X	6.1 10^−5^	6.8	13.1

**Table 5:** Wettability of coatings.

Sample	Contact Angle (°)	Contact Angle Hysteresis (°)
PEO coating	45.3 ± 1.2	–
CC 1X	146.5 ± 0.5	–
CC 2X	153.9 ± 1.4	9.7 ± 0.3
CC 3X	152.3 ± 0.9	11.3 ± 0.8

## Data Availability

Data available upon request.
